# Treatment of Scaphoid Nonunion: Radiologic Outcome of 286 Patients in 10 Years

**Published:** 2019-03-15

**Authors:** Patrick Jaminet, Marilena Götz, Phillipp Gonser, Hans-Eberhard Schaller, Oliver Lotter

**Affiliations:** ^a^Department for Plastic and Aesthetic Surgery, Hand Surgery, St. Marienhospital Borken, Borken, Germany; ^b^Department for Plastic and Hand Surgery, Diakonie Klinikum Stuttgart, Stuttgart, Germany; ^c^HNO University Hospital, Tübingen, Germany; ^d^Department of Hand, Plastic and Reconstructive Surgery, BG Trauma Center Tübingen, Tübingen, Germany; ^e^Department for Plastic, Aesthetic and Hand Surgery, Klinikum Tuttlingen, Tuttlingen, Germany

**Keywords:** scaphoid nonunion, pseudarthrosis, vascularized bone graft, union rate, bone healing

## Abstract

**Background:** We analyzed the radiologic outcome of different treatment options for scaphoid nonunion. The results were compared with literature, and a treatment algorithm was proposed. **Methods:** On the basis of a retrospective case-control study, 286 patients suffering from scaphoid nonunion were treated over a 10-year period. Patients were grouped depending on the location of the nonunion: proximal (*n* = 126), middle (*n* = 130), or distal (*n* = 30) third. In the presence of an avascular proximal fragment or after prior unsuccessful operation, interposition of a vascularized pedicled bone graft from the distal radius was performed (*n* = 82). Scaphoid healing was detected by conventional radiography and computed tomography. **Results:** Excellent healing rates of 96.3% were obtained for middle and distal third scaphoid nonunions by conventional iliac crest bone grafting (*n* = 137). Furthermore, we achieved healing rates of 91.3% for persistent nonunions using a palmar vascularized bone graft from the distal radius after prior unsuccessful operation (*n* = 23). When using a dorsal vascularized bone graft from the distal radius, scaphoid consolidation was reached in 81.1% for avascular proximal fragments (*n* = 59). **Conclusions:** Applying a sophisticated treatment algorithm, the prognosis of scaphoid nonunion is very good.

Scaphoid fractures represent the most common type of carpal fractures. Scaphoid fracture nonunion changes wrist mechanics, which can lead to carpal collapse and secondary degenerative changes.^1^ Numerous factors can contribute to nonunion: delay in treatment, displacement, proximal pole fracture, and avascular necrosis.^2^^-^5 The main goal of scaphoid reconstruction is to obtain union with normal shape and length of the scaphoid. The accepted standard treatment of scaphoid nonunion is nonvascularized grafting with internal fixation.^6^^,^^7^ Fractures with impaired vascularity have less satisfactory results when using conventional grafting techniques.^8^ For proximal pole nonunions or avascular necrosis, different vascularized bone grafts have been described. A pedicled bone graft from the distal radius can be harvested using a dorsal^9^^-^12 or palmar^13^^-^15 approach, depending on the localization of the nonunion. Union rates with vascularized bone grafts vary in literature between 27% and 100%. In this work, we retrospectively analyzed the radiologic outcome of 286 consecutive patients with scaphoid nonunion.

## PATIENTS AND METHODS

### Patient data

A total of 286 consecutive patients with scaphoid nonunion were operated over a 10-year period in our clinic (affiliation number 2). All patients had persistent scaphoid nonunion for more than 6 months. Patients presenting with advanced carpal collapse were excluded. There were 258 male and 28 female patients. The average age was 26 years (range, 13-71 years).

**Figure 1:**
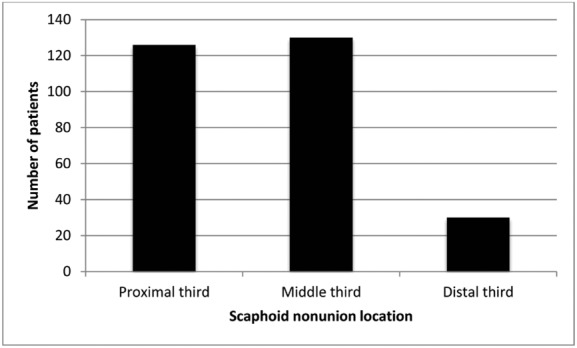
Location of the scaphoid nonunion in a total of 286 patients.

Scaphoid nonunion was located in the proximal third in 126 patients, in the middle third in 130 patients, and in the distal third of the bone in 30 patients (Fig 1). In 20 cases, a scaphoid fracture in the middle third of the bone had been previously fixed by a Herbert screw. All patients had preoperative radiographic examination (posteroanterior views in the neutral wrist position and lateral and special scaphoid views). Before the operation, informed consent was obtained from all patients.

**Figures 2-5:**
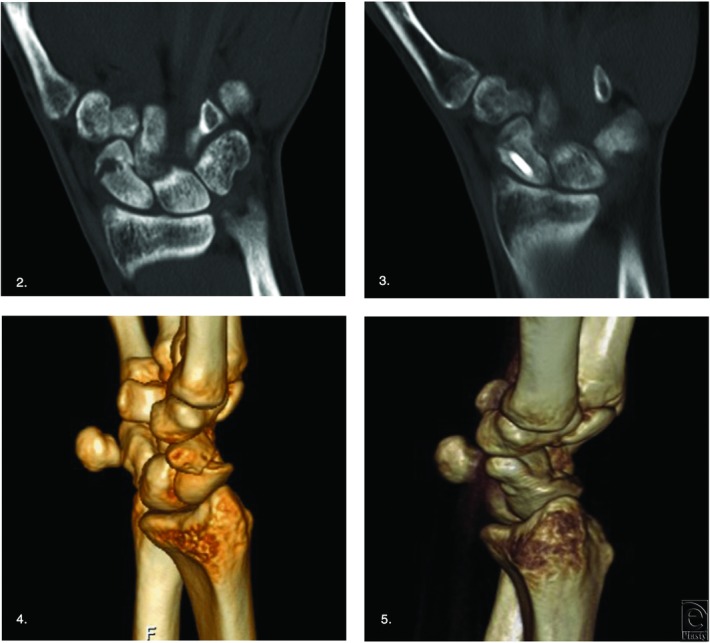
Computed tomographic and 3-dimensional computed tomographic scans of a scaphoid nonunion with corrected humpback deformity (pre- and postoperative).

Operative treatment was chosen depending on the localization of the nonunion. Distal and middle-part nonunions were reconstructed by interpositions of a nonvascularized bone graft from the iliac crest and stabilized by a Herbert screw (KLS Martin, Tuttlingen, Germany). In the case of a humpback deformity with dorsal intercalated segment instability, this situation was addressed by interposition of a structural bone graft (Figs 2-5). Persisting middle-part nonunions after unsuccessful previous operation were fixed by interposition of a palmar vascularized bone graft from the distal radius and stabilized by K-wires or, if still possible, by a Herbert screw. Proximal nonunions were fixed by interposition of a dorsal vascularized bone graft from the distal radius and stabilized by a Mini-Herbert screw (KLS Martin) when there was a complete absence of bleeding points after bony debridement. Interposition of nonvascularized bone graft was performed if bleeding was detected after releasing the tourniquet.

### Operative technique

All operations were carried out under tourniquet control and loupe magnification.

#### Dorsal approach

**Figure 6:**
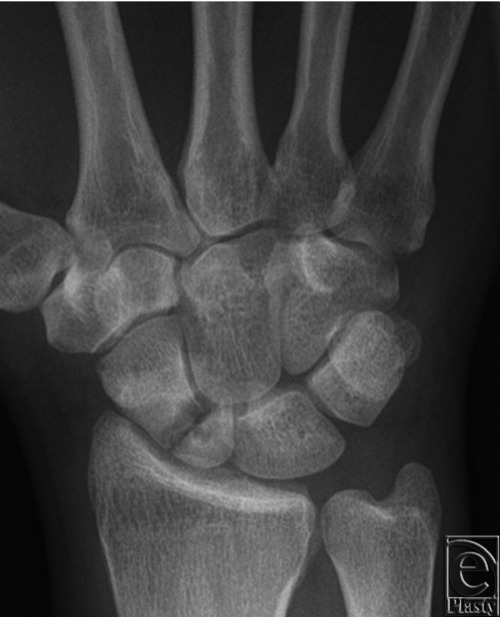
Posteroanterior radiograph showing a proximal third scaphoid nonunion.

**Figure 7:**
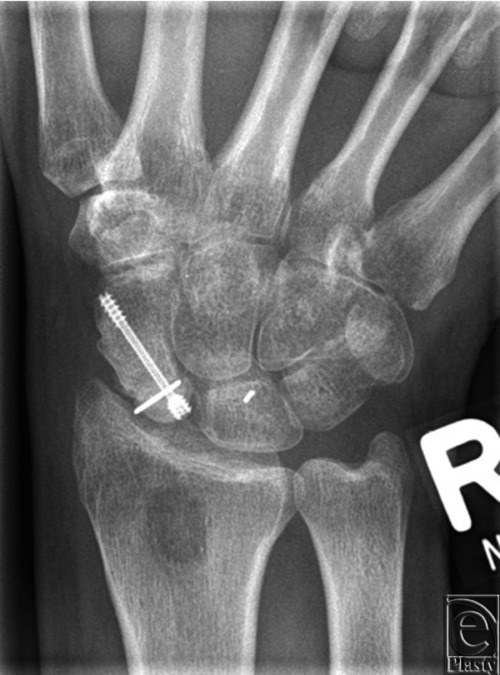
Posteroanterior radiograph showing the reconstructed and consolidated scaphoid after interposition of a dorsal vascularized bone graft from the distal radius. The graft has been additionally fixed by a 0.8-mm K-wire.

The dorsal approach was used in 123 patients. Of these patients, 59 had interposition of a dorsal vascularized bone graft from the distal radius and 64 had interposition of a nonvascularized bone graft from the distal radius or the iliac crest according to the size of the defect. After exposition of the proximal pole as described previously, radical bone debridement was performed using an osteotome.^16^ In the absence of punctuate bleeding from the debrided bone surface, a vascularized graft was raised either based on the 1,2 intercompartmental supraretinacular artery^9^ or based on a branch of the radial artery ascending deep into the first or second dorsal extensor compartments.^10^^,^^11^ Priority was given to the pedicle with the largest diameter. The graft was shaped to exactly fit into the defect. If the graft was too small, additional cancellous bone was added from the distal radius or the iliac crest. The scaphoid was then stabilized by a Mini-Herbert screw and additional K-wires if necessary (Figs 6 and 7).

#### Palmar approach

The palmar approach was used in 163 patients presenting with distal or middle third scaphoid nonunion. Of these patients, 142 had interposition of a nonvascularized bone graft from the iliac crest. Stabilization was reached by a Herbert screw as shown earlier. Twenty-three patients had undergone previous operation with persistent nonunion. In these patients, we opted for interposition of a palmar vascularized bone graft based on the palmar carpal artery as described by Mathoulin and Haerle.^14^ Stabilization was performed by K-wires or a Herbert screw, if possible.

### Outcome measures

The major outcome measure was the establishment of union with graft incorporation. Postoperative posteroanterior, lateral, and scaphoid view radiographs were obtained at 6 and 12 weeks, as well as computed tomographic scans at 12 weeks. Scaphoid union was diagnosed if bony trabeculae crossed the proximal and distal junction site of the bone graft. Radiologic imaging was evaluated by a musculoskeletal radiologist.

## RESULTS

The results are presented according to the localization of the scaphoid nonunion.

**Figure 8:**
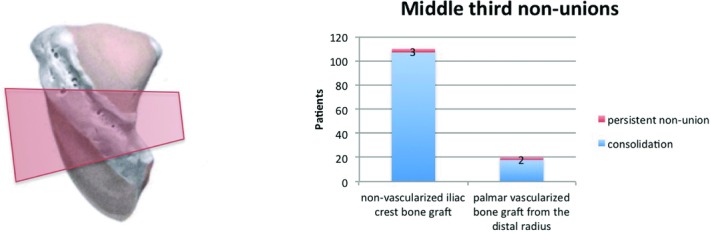
Radiologic outcome of 130 middle third nonunions (nonvascularized iliac crest bone graft vs palmar vascularized bone graft from the distal radius).

In 130 patients, scaphoid nonunion was located in the middle third. Of these patients, 110 underwent primary interposition of a nonvascularized bone graft from the iliac crest after radical debridement. Bony union was diagnosed in 97.3%. The remaining 20 patients had scaphoid nonunion after previous operation. These nonunions were fixed by interposition of a palmar vascularized bone graft from the distal radius. Union was obtained in 18 patients after 12 weeks (90%) (Fig 8).

**Figure 9:**
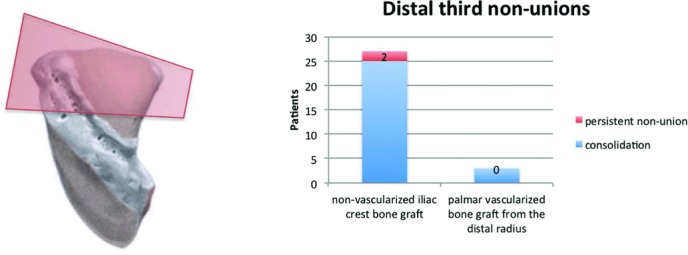
Radiologic outcome of 30 distal third nonunions (nonvascularized iliac crest bone graft vs palmar vascularized bone graft from the distal radius).

In 30 patients, scaphoid nonunion was located in the distal third. In 27 patients, scaphoids were primarily reconstructed by interposition of a nonvascularized bone graft from the iliac crest. In total, 92.6% of the scaphoids healed. For the remaining 3 patients who suffered from nonunion after previous operation, union was obtained after 12 weeks by interposition of a palmar vascularized bone graft from the distal radius (Fig 9).

**Figure 10:**
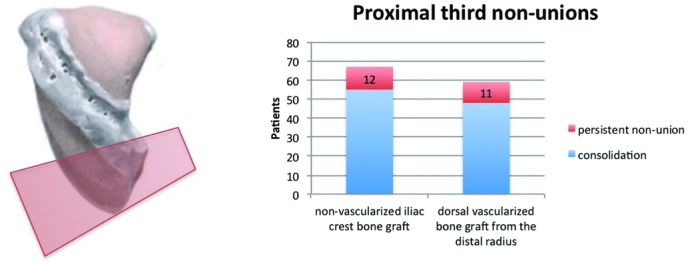
Radiologic outcome of 126 proximal third nonunions (nonvascularized iliac crest bone graft vs dorsal vascularized bone graft from the distal radius).

In 126 patients, scaphoid nonunion was located in the proximal third. In 67 patients, the scaphoid was reconstructed by interposition of a nonvascularized bone graft from the iliac crest if intraoperative bleeding was detected from the proximal pole. Scaphoid union was diagnosed in 82.1% of these cases. In the absence of intraoperative bleeding, scaphoid reconstruction was performed by interposition of a dorsal vascularized bone graft from the distal radius in 59 patients, obtaining union in 81.1% at 12 weeks (Fig 10).

## DISCUSSION

The optimal treatment of scaphoid nonunion remains a complex problem. The healing potential of the scaphoid strongly depends on vascularity and stability/compression. Unstable and proximal scaphoid nonunions have been associated with decreased rates of bony healing after nonvascularized bone-grafting procedures.^3^^,^^17^^,^^18^ One of the main problems is the vascular impairment of the proximal segment of the scaphoid.^11^^,^^19^ Green^20^ reported a union rate of 0% if there was no intraoperative bleeding out of the proximal segment. While a recent literature review reports overall union rates between 74% and 84%, this number is by far lower in proximal pole pathologies.^21^^,^^22^ To improve clinical outcome, vascularized pedicled grafts from the distal radius were introduced with reported union rates of up to 100%.^10^^,^^12^^,^^14^^,^^23^^-^27

In our study, conventional bone grafting from the iliac crest was performed in nonunions of the distal and middle third (without any previous operation) as well as in nonunions of the proximal third if there was intraoperative bleeding out of the proximal pole. We could show bony healing rates in 97.3% (middle third), 92.6% (distal third), and 82.1% (proximal third). For the latter, nonunions were persistent in almost 20% of our patients despite the presence of intraoperative bleeding. Green^20^ achieved consolidation in 92% of cases if intraoperative bleeding out of the proximal pole was seen.

Megerle et al^16^ reported on the treatment of scaphoid nonunions in the proximal third with conventional bone grafting and Mini-Herbert screw fixation despite the vascularity of the proximal pole. Of 31 patients in their study, bone union was obtained in only 21 patients (67.7%).

In those patients with impaired healing after primary conventional bone grafting of middle and distal third nonunions, we reconstructed the scaphoid by interposition of a palmar vascularized bone graft from the distal radius. The union rate that we obtained for these patients (*n* = 23) was 91.3%. Mathoulin and Haerle^14^ reported a union rate of 100%. Seventeen patients were included in this study, but only 10 of them had prior unsuccessful operation, which could be the reason for this excellent union rate.

In the absence of intraoperative bleeding out of the proximal pole, we reconstructed the scaphoid by interposition of a dorsal vascularized bone graft from the distal radius either based on the 1,2 intercompartmental supraretinacular artery^9^ or based on a branch of the radial artery ascending deep into the first or second dorsal extensor compartments.^10^^,^^11^ We reached union rates of 81.1%, which is high compared with the first describers of the technique. Straw et al^9^ showed union rates of 12.5% (2/16 patients) in the presence of proximal pole avascularity. Boyer et al^11^ showed union rates of 60% (6/10 patients). Both authors reported dissatisfaction with the technique and go as far as not recommending it for treatment of proximal pole necrosis. Zaidemberg et al^10^ used the dorsal vascularized bone graft in 11 patients in long-standing pseudarthrosis of the scaphoid but without specifying the localization of the nonunion.

Malizos et al^23^ reported on 20 proximal third nonunions managed with dorsal radius bone graft and 10 waist nonunions managed by palmar radius bone graft. They found an overall healing rate of 100% and concluded that vascularized bone grafts (palmar or dorsal, depending on the localization) are reliable and efficient.

Other authors suggest a free vascularized medial femoral condyle bone graft after failed scaphoid nonunion surgery^28^ with good results.

In our series, we present healing rates in 286 patients, which are, compared with other studies, a very high number, including 126 proximal third nonunions. Of these 126 patients, 59 showed avascular proximal pole necrosis demonstrated by lack of intraoperative bleeding. Pre- and postoperative magnetic resonance imaging was not standardized, the main attention being turned to intraoperative bleeding out of the proximal pole.

Stability and vascularity are the most important factors that govern the healing of nonunion. Both factors were simultaneously addressed in our large series, containing an overall number of 82 vascularized radial bony grafts.

## CONCLUSIONS

**Figure 11:**
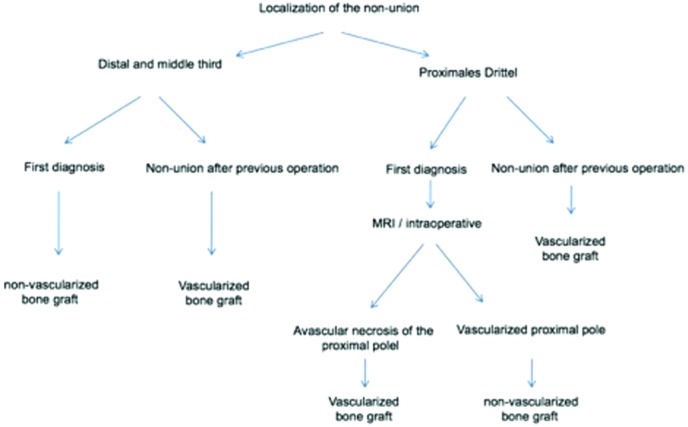
Algorithm for treatment of scaphoid nonunion. MRI indicates magnetic resonance imaging.

We present our treatment algorithm (Fig 11) for scaphoid nonunion, including the localization of the nonunion and the possible causes for unsuccessful prior treatment. Using this model, we can achieve a high rate of bone healing if the experienced surgeon offers the required microsurgical skills to properly raise the vascularized bone grafts from the distal radius.^29^
